# Optimizing Scarce Resource Allocation During COVID-19: Rapid Creation of a Regional Health-Care Coalition and Triage Teams in San Diego County, California

**DOI:** 10.1017/dmp.2020.344

**Published:** 2020-09-10

**Authors:** Asha Devereaux, Holly Yang, Gilbert Seda, Viji Sankar, Ryan C. Maves, Navaz Karanjia, John Scott Parrish, Christy Rosenberg, Paula Goodman-Crews, Lynette Cederquist, Frederick M. Burkle, Jennifer Tuteur, Chiara Leroy, Kristi L. Koenig

**Affiliations:** Sharp Coronado Hospital, Coronado, California; San Diego County Medical Society, Scripps Health, San Diego, California; Naval Medical Center San Diego, San Diego, California; Kaiser Permanente, San Diego, California; University of California-San Diego Health, San Diego, California; Be There San Diego, San Diego, California; Harvard Humanitarian Initiative, Harvard University & T.H. Chan School of Public Health, Boston, Massachusetts; County of San Diego, Health and Human Services Agency

**Keywords:** health-care rationing, mass casualty incidents, triage, capacity building, disaster medicine

## Abstract

Successful management of an event where health-care needs exceed regional health-care capacity requires coordinated strategies for scarce resource allocation. Publications for rapid development, training, and coordination of regional hospital triage teams to manage the allocation of scarce resources during coronavirus disease 2019 (COVID-19) are lacking. Over a period of 3 weeks, over 100 clinicians, ethicists, leaders, and public health authorities convened virtually to achieve consensus on how best to save the most lives possible and share resources. This is referred to as population-based crisis management. The rapid regionalization of 22 acute care hospitals across 4500 square miles in the midst of a pandemic with a shifting regulatory landscape was challenging, but overcome by mutual trust, transparency, and confidence in the public health authority. Because many cities are facing COVID-19 surges, we share a process for successful rapid formation of health-care care coalitions, Crisis Standard of Care, and training of Triage Teams. Incorporation of continuous process improvement and methods for communication is essential for successful implementation. Use of our regional health-care coalition communications, incident command system, and the crisis care committee helped mitigate crisis care in the San Diego and Imperial County region as COVID-19 cases surged and scarce resource collaborative decisions were required.

San Diego County is the fifth largest county in the United States (US), with a population approximating 3.3 million people. San Diego County public health officials gained early management experience with the coronavirus disease 2019 (COVID-19) pandemic as the site for quarantine of US citizens evacuated from Wuhan and cruise ship passengers to Marine Corps Air Station Miramar, which required some local hospitalizations in early February 2020.^1^

When the first San Diego County resident with COVID-19 was identified on March 9, 2020,^2^ news reports of rapidly rising death rates, infected health-care workers, and health system capacity issues in China, Italy, and Washington State, accelerated our preparedness activities. In collaboration with hospital and operational leadership, community health centers, the Hospital Association of San Diego and Imperial Counties (HASD&IC), and the San Diego County Medical Society (SDCMS), San Diego adopted a 3-pronged strategy for COVID-19 management.^3^ The framework was to: (1) flatten the curve (by means of social distancing); (2) enhance health-care system capacity^4^; and (3) use an Incident Command System to coordinate and manage resources. San Diego leaders additionally recognized the need to develop a system for Crisis Care that would be ethical and equitable throughout the region in the event that the pandemic evolved to the point where local population health needs exceeded available resources.^5^ This study describes the process for rapid development of a regional collaborative health-care coalition to optimize scarce resource allocation during COVID-19. Other communities may benefit from the key principles used to create the coalition, the step-by-step processes used to rapidly achieve consensus on a complex topic, ways to include a diverse group of participants, use of virtual communication platforms, and methods for education.

## CHALLENGES AND OBJECTIVES

1. Establish a county-wide mechanism for shared resource allocation, multi-stakeholder vetting, and a communication process for the establishment of Crisis Care.
2. Rapidly educate local, community, and hospital systems about the steps necessary for a systematic process to those unfamiliar with the prior Institute of Medicine/National Academies (IOM/NAM) or state frameworks.^6^
3. Complete the above in an abbreviated timeframe (ie, several weeks) during an environment of rapid regulatory change and evolving science surrounding COVID-19.
4. Communicate virtually due to COVID-19 isolation restrictions.
5. Create Triage Teams and provide training tools for hospital systems.

## TIMELINE

**FIGURE 1:**
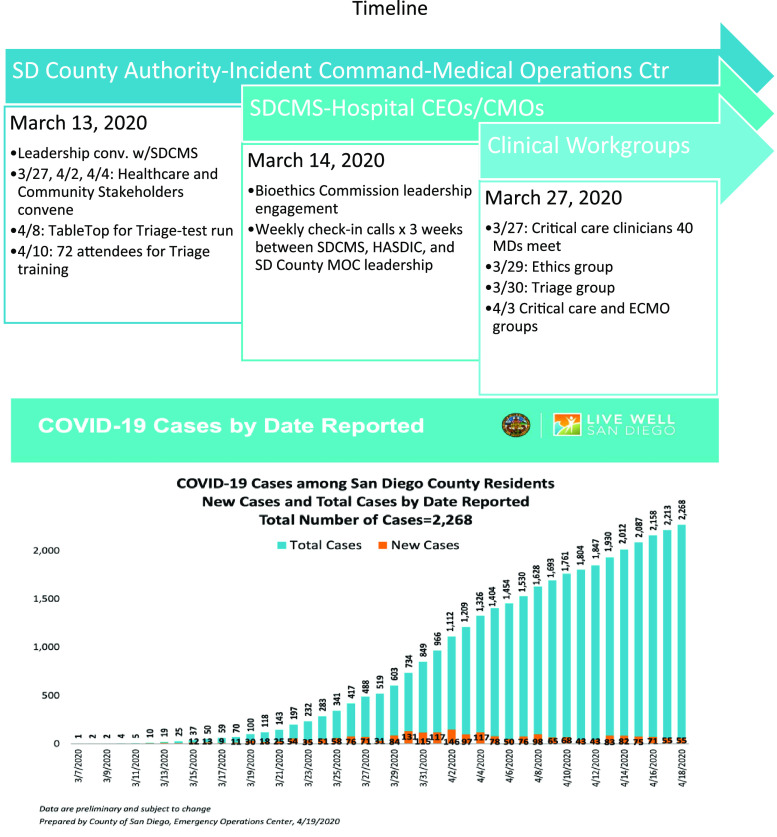
Timeline.

On March 11, 2020, when the World Health Organization (WHO) declared COVID-19 a pandemic and due to the accelerating concerns witnessed around the world, the San Diego County Medical Society and the San Diego Bioethics Commission engaged the County health officials regarding plans for the potential allocation of scarce resources during a surge of patients (Figure 1). By mid-March, it was determined that practical guidance from the federal government was unlikely, and that solutions and authority would need to be developed locally with community resources and values considered. The global scarcity of personal protective equipment (PPE) for health-care workers and limited severe acute respiratory coronavirus 2 (SARS-CoV-2) testing triggered conservation strategies in San Diego and served to highlight the need for Crisis Care preparedness. Therefore, various local leaders and San Diego-based experts from the Taskforce for Mass Critical Care partnered to draft a Community Standard of Care Consensus document for Crisis Care during COVID-19.

## DEVELOPMENT OF HEALTH-CARE COMMUNITY COALITIONS

As originally described by Courtney et al. in 2009, the formation of Health-Care Coalitions is vital to respond to a mass critical care event and share resources in a coordinated manner.^7^ The San Diego area maintains collaborative relationships through health system disaster coordinators and the county; however, intensivists are not commonly involved in regional planning and meeting activities, and few physicians can leave patient care responsibilities to prepare for what may be a “never-event.”^8^ This has resulted in a lapse in knowledge of the formation of health-care coalitions, roles, responsibilities and the Incident Command System (ICS). Using some of the updated framework by the American College of Chest Physicians and the Society of Critical Care Medicine, multiple members worked simultaneously to engage various stakeholders.^9-11^ Following US national health-care preparedness guidance,^6,12^ our approach also included coordination with the regional ICS. The San Diego ICS involves both an Emergency Operations Center (EOC) and a Medical Operations Center (MOC). Once it was determined that the County elected leadership and the Public Health Officer would be the declaration authority if state or federal declaration was absent for Crisis Care, health-care systems’ alignment rapidly formed.

## STAKEHOLDERS NECESSARY FOR RAPID REGIONAL COALITIONS

**FIGURE 2:**
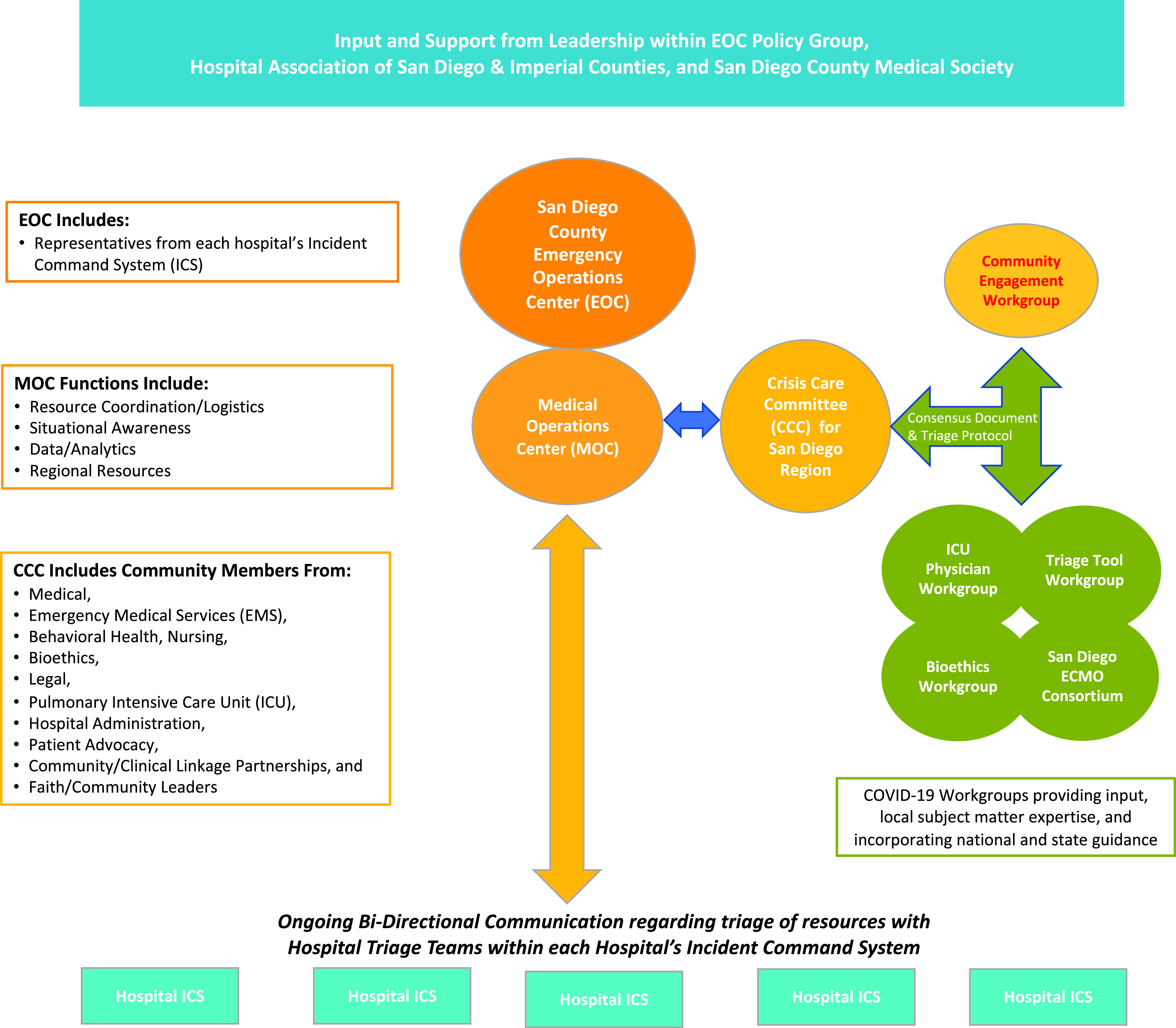
Organization Chart for Regionalization.

A core group of 4 individuals was able to initiate the framework for coalition and workgroup formation (Figure 2). Organizers ensured representation from all health-care systems throughout the county. This framework includes:

- Administrative support from **Regional Public Health/Government Authority**
- Leadership from **Professional Medical Society** representatives
- Engagement of each hospital **Chief Medical Officer and Chief Executive Officer** during weekly (virtual) meetings and triage team training^13,14^
- Selection and formation of **Intensive Care Unit (ICU), Ethics, Triage, and Extracorporeal Membranous Oxygen (ECMO)** workgroups designated by leaders from each hospital system, including the Veteran’s Administration (VA), Kaiser, UC San Diego, and the US Navy
- Formation of a **Community Crisis Care Committee**. Representing San Diego area’s Pulmonary, Critical Care, Hospitalists, Out-Patient, Emergency, and Pediatric Medicine, Behavioral/Wellness/Mental Health, Community Health Advocates, Clergy, Government, Hospital Administration, SDCMS Leaders, Medicaid, Nursing, Bioethics, Palliative Care, Hospice, Veterans, Homeless, and Legal Services. Several members represented more than 1 sector. This committee met weekly.

Workgroup and Committee members were solicited by means of email requests to area hospital leaders and disaster experts. Once key members were identified, word rapidly spread regarding the County’s collaborative work and nearly every hospital in the region became engaged. Recommendations from workgroups were discussed with the Crisis Care Committee for feedback regarding fairness and elimination of bias. Throughout this week of planning, teams were informed by, and rapidly adapted to, national and international events as well as evolving ethical debates about scarce resource allocation.^15^ There was enthusiastic engagement from all stakeholders who attended videoconferences, frequently lasting over 2 h each. Plans and resource adaptation ideas were freely exchanged among institutions, reflecting the trust provided by leadership authorities toward the workgroups. Mutual trust, transparency, and respect are a necessary requirement for effective coalition development and cannot be understated. During this busy period of activity, short-notice meetings were frequent and at times neglected the hospital ICS (HICS). This early weakness was improved by use of redundant communication and providing advanced notification to workgroup members and executives of meetings.

## TRIAGE OF SCARCE RESOURCES—TRIAGE TEAMS

To ensure resources are shared fairly in the setting of scarcity, it is essential that the triage process for patients requiring critical care resources be consistent across all hospital systems, and includes access to relevant subspecialty expertise. The goals of crisis care triage shift the standard practice of individual patient considerations to a broader population-based public health to extend scare critical resources to ensure the survival of the largest possible number of patients. Identifying patients who might need critical care resources and defining their individual risks for mortality become key to maximize the greatest good for the greatest number.^15-17^ The shift to population-based health triage as a concept is only used when a health system’s ability to surge has been maximized and exceeded.

To implement Crisis Care and triage of scarce resources, committee organizers established Triage Teams and created Job Action Sheets within HICS.^18^ The following principles were instrumental in the development and formation of Triage Teams.

1. Health-care systems have triage officers with the following qualities: (a) critical care triage training to provide optimum allocation of resources; (b) situational awareness at both a regional and institutional level; (c) experience in care of the patient being triaged; (d) individual triage officers or teams consisting of a senior critical care physician and an acute care (critical care-experienced) physician or nurse be designated to make mass critical care triage decisions in accordance with previously prepared, publicly vetted, and widely disseminated guidelines.
2. A vetted triage protocol (SD Crisis Consensus Care Draft document), rather than clinical judgment alone, will be used.
3. Health-care systems will leverage technology in triage to augment clinical assessment in an effort to improve incremental survival and efficiency of resource allocation.
4. The triage decision process will have an appeals mechanism in case of deviation from an approved process (which may be a prospective or retrospective review) or a clinician request for reevaluation in light of novel or updated clinical information (prospective).
5. Intensivists should leverage telemedicine and other technologies when possible to assist those health-care systems with few or no intensivists to help with triage.
6. There will be legal protections for providers conducting triage in a disaster as well as a psychological support system for triage officers and health-care providers. When legal protection is not possible, providers need to follow acceptable, consensus-based community standards of care pertaining to triage in mass critical care.
7. Intensivists should work within their local and regional health-care system to determine the plan for augmentation of critical care resources to care for as many patients as possible. Resources should be shared across geographic regions to maximize lives saved.
8. Triage is a dynamic process that may change rapidly based on available resources, knowledge of the disease process, patient volume, and acuity.

**FIGURE 3:**
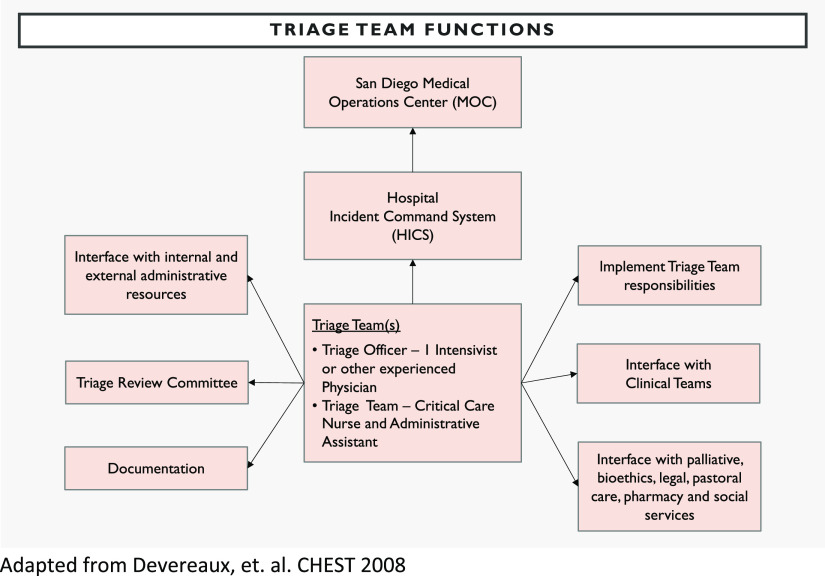
Triage Team Development and Education.

The biggest challenge in creating a triage algorithm was the substantial gap in the literature as to how to prognosticate accurately regarding COVID-19.^19^ Because triage is designed to be used on all patients, it was, therefore, essential to have subspecialty expert input (eg, from trauma, emergency medicine, neurocritical care, ethics, and medical intensivists) to ensure fairness for all clinical conditions is built into the triage algorithm. Nonstandardization of hospital medical record systems and variable personnel resources created additional challenges in creating a uniform and unbiased triage team. This was overcome by sharing technological solutions, providing written protocols, and training as an entire county system. All San Diego hospitals identified triage personnel that would be on-call, should a crisis level of surge be declared and crisis standard of care triage need to be implemented (Figure 3).

## SITUATIONAL AWARENESS

To create a system that would permit Triage Teams to share information, decisions, and allocate resources, a transparent dashboard visible to all facilities in the region is required to facilitate real-time decision-making. This provides situational awareness and reduces the moral distress that may be experienced by health-care providers with creating and executing such processes.

The ICU workgroup identified the following areas requiring development:

1. A system to track and report resources in real-time at a countywide level
2. A resource allocation process that is harmonized across multiple hospital systems
3. Harnessing electronic health record systems to facilitate triage scoring
4. Identifying infrastructure for communication regarding this triage process within and across hospital systems
5. Just-in-Time education processes for hospital staff, providers, patients/families, and the public regarding triage

Since March 2020, each hospital has provided data regarding staff, patient census, PPE, and ventilator supply to the County Emergency Medical Services electronic data collection system (Resource Bridge module of Image Trend product). Although each hospital had no visibility of other hospitals’ data, each had seen the aggregate county-wide data during this pandemic and previous surge conditions from influenza and fire seasons. Using the currently available Emergency Medical Services (EMS) data, we created a data workgroup that requested a red-yellow-green display of Staff, Stuff, and Structure to visualize resource capacity. This permits the MOC and Crisis Care Committee Members to determine at a glance if the supply of resources is adequate, or to quickly focus on areas that are low or critically low. Unique to this pandemic, hospital triage officers, CEOs, and CMOs requested to view others’ data, as well as share their own in real time. To achieve the mission, they insisted not only to know the status of their own hospital’s resources, but also to be aware when their neighboring hospital was beginning to run low, so they could prepare to share their own resources.

## EDUCATION AND TRAINING

Tabletop exercises are commonly used by emergency management for face to face simulated education of staff roles and responsibilities during disasters. As it pertains to triage education, a tabletop exercise allows the creation of a nonthreatening environment where new triage officers (Figure 3) can rehearse their roles, ask questions, and troubleshoot potential problems with the triage protocol. Unique to COVID-19, hospital leadership and clinicians involved in triage during a pandemic will need just-in-time education and training long before any triage protocol is implemented. It is important to prepare triage officers to understand the rationale and ethical principles in triage, provide training opportunities to implement the triage protocol, and to have a process in place for real-time revision of the protocol as understanding of the disease unfolds. Our education and training was complicated by the need to train triage officers from many different hospitals while maintaining social distancing. Therefore, we used a web-based platform for video and audio conferencing.

In preparing triage officers from area hospitals, San Diego conducted virtual tabletop exercises using Zoom. Our training objectives were to review the regional criteria for transitioning from first-come-first-served patient care to a triage process during crisis standards of care, review the roles and responsibilities of triage officers, practice using the triage protocol in 5 case scenarios, and to use feedback from the case scenarios to further refine the triage protocol. Using Zoom’s virtual break-out rooms, San Diego County successfully conducted tabletop exercises for more than 70 participants at a time, representing all San Diego acute care hospitals and integrated health systems. In addition to a successful training exercise, this permitted the identification of a challenge within the triage protocol, which was able to be remedied through a small modification by the Triage Tool workgroup. The exercises facilitated greater exposure of the triage protocol to the intended audience and resulted in a better instrument.

## COMMUNICATION WITH THE PUBLIC AND WITH PATIENTS/FAMILIES

To achieve the ethical responsibility of transparency, a subgroup was formed from the Crisis Care Committee to create a communication plan for the public in the event of scarce resource allocation. The goal of this endeavor was to mitigate fear, encourage continued social distancing, and offer assurance to the public that the county and community was actively involved in planning for contingency and crisis. This subgroup created a handout for distribution to patients/families if admitted to a hospital when Crisis Standards are invoked. In executing the ethical duties of distributive and procedural justice, the goal of this undertaking is to ensure that every person admitted to any hospital in the county will receive the same information and be subject to the same allocation criteria regardless of the particular hospital^20^ (see an example in the Appendix). Due to the separation of patients from family members for safety during COVID-19, the challenge of providing information to patients and family members has been considered and will require duplication of staff efforts and multi-media publication efforts.

## SUCCESSFUL AND CONTINUED MITIGATION

As a result of the collaborative process in formation of clinical workgroups, hospital systems communication, and working within the SD County Incident Command System, the Crisis Care Committee and health-care coalitions have been able to assist with the initial allocation of remdesivir provided by means of the federal government^21^ and to provide a neighboring county that evacuated over 500 patients with protocols based upon the work done in San Diego. As COVID-19 cases accelerated in Southern California, the ECMO workgroup activated, sharing protocols and agreements. The hospital systems’ coalitions, led by hospital chief medical officers and chief executive officers have now assumed the responsibilities for Crisis Care in San Diego following release of California State guidelines in late June 2020.^22^ The work done by the Crisis Care Committee of San Diego laid the foundation for resource sharing which has mitigated scarce resource situations.

## CONCLUSION

Within a span of 3 weeks, San Diego County was able to implement a triage allocation process and form health-care coalitions in response to the COVID-19 pandemic crisis. As the surge of COVID-19 patients outstrips medical resources throughout the world, the need for health-care systems to work together with public health authorities to share scarce resources is more important than ever. Although national and state/regions may develop guidelines for allocation of scarce resources, implementation is at the hospital, clinician, and patient bedside level. This requires changes in communication by means of an incident command system, understanding the concept of triage, and development of transparent situational awareness for providers, the public, government, and health-care systems. To date, only 23 U.S. states have Crisis Care guidelines, and our work was formed during a time when California was developing and revising the state protocol. By addressing this gap, we realized that the process of community, hospital systems, providers, and public health engagement resulted in open and frank communication, establishment of trust and transparency, a pathway to sharing resources, and a method for continuous improvement. Plans for nephrology, pediatrics, and more detailed community engagement are actively being addressed. This dynamic process will be sustained and will provide the foundation for ongoing COVID-19 and other disaster response activities in the region.

## United States Government Employees or Military Service Members

Drs Seda, Maves, and Parrish are United States government employees or military service members. This work was prepared as part of their official duties. Title 17 U.S.C. §105 provides that “Copyright protection under this title is not available for any work of the United States Government.” Title 17 U.S.C. §101 defines a U.S. Government work as a work prepared by a military service member or employee of the U.S. Government as part of that person’s official duties. The opinions and assertions contained herein are those of the authors and do not reflect the official views or position of the Department of the Navy, Department of Defense, Department of Veterans Affairs, the United States Government, nor of the academic institutions with which the authors are affiliated.

## Supplementary material

For supplementary material accompanying this paper visit http://dx.doi.org/10.1017/dmp.2020.344. click here to view supplementary material
