# Supplementary material for: Optimizing Scarce Resource Allocation During COVID-19: Rapid Creation of a Regional Health-Care Coalition and Triage Teams in San Diego County, California
Source: Disaster Med Public Health Prep. 2020 Sep 10:1–7. doi: 10.1017/dmp.2020.344 (PMC7684024; doi:10.1017/dmp.2020.344)
Supplement: Supplementary file 1 [file S1935789320003444sup001.docx]

**Life Support During the COVID Pandemic**


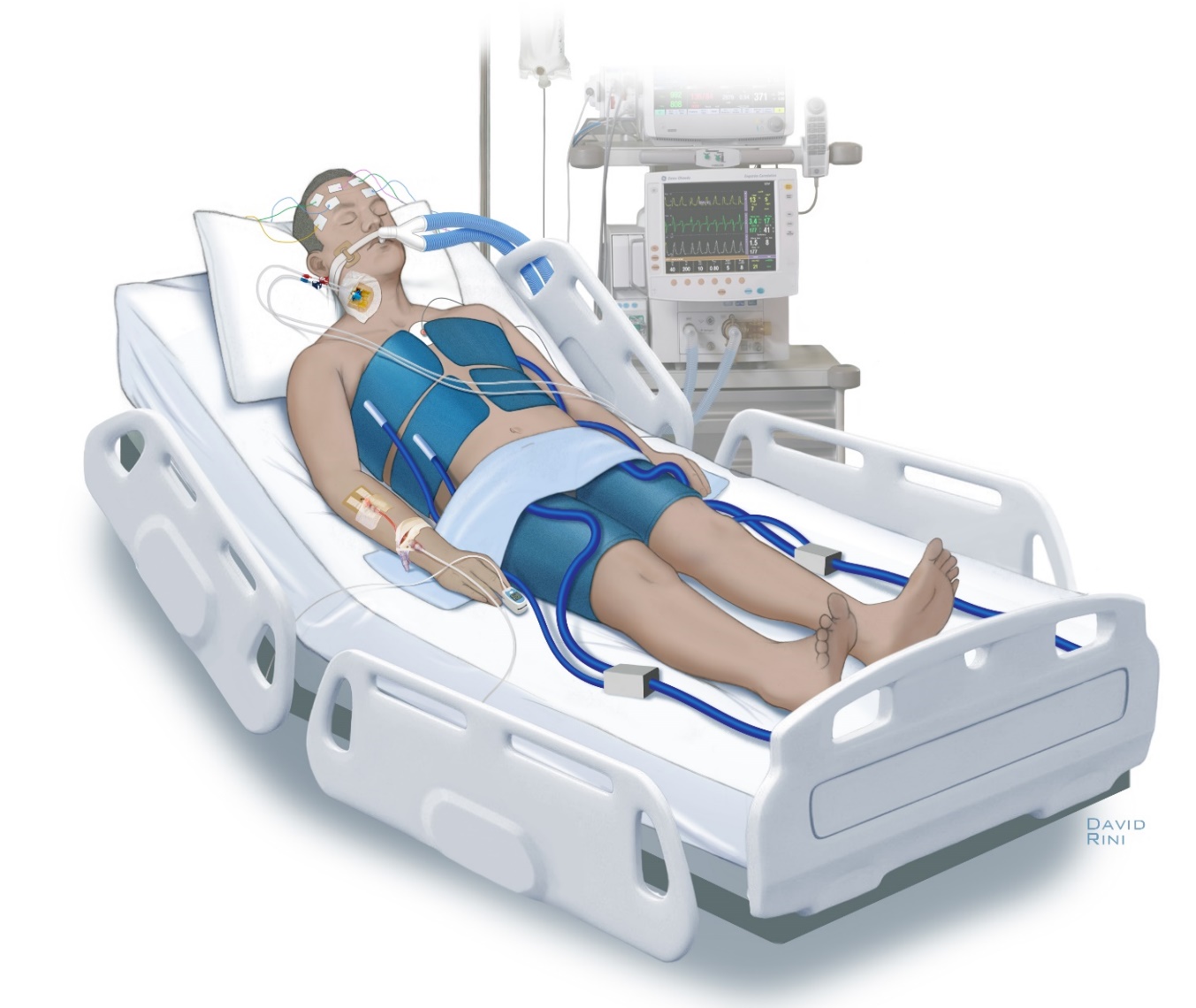


*Image used with permission from David Rini*

This is an unusual time, with very large numbers of very sick people right now. Some people are getting so sick that they need a life support machine (for example, a ventilator or breathing machine – see picture).

Because of the current pandemic, there might not be enough life support machines/intensive care (“ICU”) for everyone who needs them. Hopefully, this does not happen.

In this very difficult time, it’s really important to be clear about your values and main concerns for your health care.

**How would decisions about who gets a life support machine/intensive care (“ICU”) be made?**

If there is a shortage, a team of doctors and other medical professionals will review all cases of patients who need life support machines. There may not be enough life support machines for all who want one. This team will make tough decisions based on the best medical information available and the patient’s clinical condition. The team will not be given information about age, race, ethnicity, religion, disability status, insurance, or other unrelated things.

**What are my choices?**

This is an important time to think about what you would want. People often have thoughts about life support machines.

- Some people say, *“I would want to have a life support machine if there is one.”*
- Other people may say, “*I would not want any kind of life support or breathing machine. If it comes to that, please keep me comfortable and let me have a natural death.”*

Your health care team **and your loved ones** need to know what you want if you need a life support machine/intensive care (“ICU”). Please talk to them about what you would want.

All patients admitted to the hospital will receive medical care. For people who do not get life support machines/intensive care (“ICU”), other available treatments will be offered including the relief of pain and suffering.

**What are the next steps?**

Even if you do not know the answers to the above questions right now, you should:

- Choose someone who will help make your medical decisions. This is the person who speaks for you if you cannot speak for yourself
- If you already have an advance directive, a medical care power of attorney, or Physician Order for Life Sustaining Treatment (POLST), please provide a copy to your health care team.
- Talk to this person so they know what is truly important to you. **This is the most important step.**
- Make sure to tell your doctor who this person is.
- As your condition changes, you may change your mind about the kind of treatments you want and don’t want. Let your health care team know of any change so your wishes can be respected.
- *Should you or your advocate disagree with the determination made by your doctors, you can request to have that determination reviewed by an independent group of medical professionals.*

This is a hard time for everyone. We’re all working together. Please continue this conversation with your medical team with any questions you may have.
